# Transcriptional Comparison of Human and Murine Retinal Neovascularization

**DOI:** 10.1167/iovs.64.15.46

**Published:** 2023-12-28

**Authors:** Laurenz Pauleikhoff, Stefaniya Boneva, Myriam Boeck, Anja Schlecht, Günther Schlunck, Hansjürgen Agostini, Clemens Lange, Julian Wolf

**Affiliations:** 1 Eye Center, Medical Center – University of Freiburg, Faculty of Medicine, University of Freiburg, Freiburg, Germany; 2 Department of Ophthalmology, Amsterdam University Medical Centers, Amsterdam, the Netherlands; 3 Department of Ophthalmology, Boston Children's Hospital, Harvard Medical School, Boston, Massachusetts, United States; 4 Institute of Anatomy and Cell Biology, University of Würzburg, Würzburg, Germany; 5 Eye Center at St. Franziskus Hospital, Münster, Germany; 6 Molecular Surgery Laboratory, Department of Ophthalmology, Byers Eye Institute, Stanford University, Palo Alto, California, United States

**Keywords:** oxygen-induced retinopathy, OIR, proliferative diabetic retinopathy, PDR, retinal neovascularization, RNV, RNA sequencing, disease animal models, mouse, human, diabetes mellitus

## Abstract

**Purpose:**

Retinal neovascularization (RNV) is the leading cause of vision loss in diseases like proliferative diabetic retinopathy (PDR). A significant failure rate of current treatments indicates the need for novel treatment targets. Animal models are crucial in this process, but current diabetic retinopathy models do not develop RNV. Although the nondiabetic oxygen-induced retinopathy (OIR) mouse model is used to study RNV development, it is largely unknown how closely it resembles human PDR.

**Methods:**

We therefore performed RNA sequencing on murine (C57BL/6J) OIR retinas (n = 14) and human PDR RNV membranes (n = 7) extracted during vitrectomy, each with reference to control tissue (n=13/10). Differentially expressed genes (DEG) and associated biological processes were analyzed and compared between human and murine RNV to assess molecular overlap and identify phylogenetically conserved factors.

**Results:**

In total, 213 murine- and 1223 human-specific factors were upregulated with a small overlap of 94 DEG (7% of human DEG), although similar biological processes such as angiogenesis, regulation of immune response, and extracellular matrix organization were activated in both species. Phylogenetically conserved mediators included *ANGPT2, S100A8, MCAM, EDNRA,* and *CCR7*.

**Conclusions:**

Even though few individual genes were upregulated simultaneously in both species, similar biological processes appeared to be activated. These findings demonstrate the potential and limitations of the OIR model to study human PDR and identify phylogenetically conserved potential treatment targets for PDR.

Retinal neovascularization (RNV) is the common hallmark of ischemic retinopathies, such as ischemic retinal vein occlusion (RVO), proliferative diabetic retinopathy (PDR), and retinopathy of prematurity (ROP), which can lead to transient or irreversible loss of vision in all age groups, from infants to seniors. In particular, diabetic retinopathy (DR) is a leading cause of vision loss, globally affecting more than one third of the 295 million diabetic patients worldwide.^1^ With its incidence predicted to rise even further, prevention and treatment of the sight-threatening consequences of DR are of particular interest.^2^

Pathophysiologically, RNV represents a common terminal pathway as a consequence of a similar series of events in ROP, PDR, and RVO.^3^ During the course of these diseases, obliteration or insufficiency of small retinal vessels causes ischemia in significant parts of the retina, which in turn leads to the release of proangiogenic factors such as the vascular endothelial growth factor (VEGF). These factors promote development of RNV, which in turn can bleed (thus causing vitreous hemorrhage) or cause tractional retinal detachment, leading to transient or permanent loss of vision.^4^

In everyday clinical routine, prevention and inhibition of RNV comprise the primary treatment goal in ischemic retinopathies. Traditionally, panretinal laser photocoagulation (PRP) with a focus on ischemic areas is considered as first-line treatment.^5^ Potential side effects of this disruptive procedure, however, include areas of scotoma and delayed dark adaptation.^6^ Recently, intravitreal anti-VEGF injections have also been used to treat ROP^7^ and PDR,^8^^,^^9^ yielding promising results and subsequent FDA approval for the treatment of both diseases.^10^^,^^11^ However, because underlying ischemia persists and is not being treated causatively, injections have to be applied regularly over a long period of time and thus represent a significant treatment burden and carry an increased risk of intraocular infection. Moreover, about 30% of PDR patients show persistent neovascular activity despite treatment,^12^ indicating the need for new treatment modalities.

One of the most used mouse models to study RNV in vivo and to identify new treatment targets is the oxygen-induced retinopathy (OIR) model, where newborn mice are initially exposed to hyperoxia, causing central retinal vessel loss. Upon return to normoxia after five days,^13^^,^^14^ the relative retinal hypoxia in central parts of the murine retina induces VEGF-driven RNV development.^15^^–^^17^ Considering that this sequence of events shares many similarities with those observed in human ischemic retinopathies, the OIR model has been used extensively as an animal model to study ROP.^16^^,^^18^^,^^19^ Because current DR animal models only poorly reflect RNV development in PDR,^20^ the OIR model has also been used to model the pathophysiology of PDR.

However, it remains a matter of debate whether the eyes of young, nondiabetic murine pups adequately resemble the changes seen in adult patients with diabetes. Therefore the aim of this study was to perform an unbiased transcriptomic comparison between murine and human RNV to unveil how closely the OIR model resembles RNV in human PDR and whether phylogenetically conserved mediators of RNV formation exist that could serve as potential therapeutic targets in proliferative retinal disease.

## Methods

### Mice

Pregnant C57BL/6J female wildtype mice were purchased from Charles River, and the pups were used as experimental animals. All animal experiments were approved by the local authority (Regierungspraesidium Freiburg, Application number G17-11) and were performed in accordance with the respective national, federal, and institutional regulations. All experiments adhered to the ARVO Animal Statement.

### Oxygen-Induced Retinopathy Mouse Model

**Figure 1.**
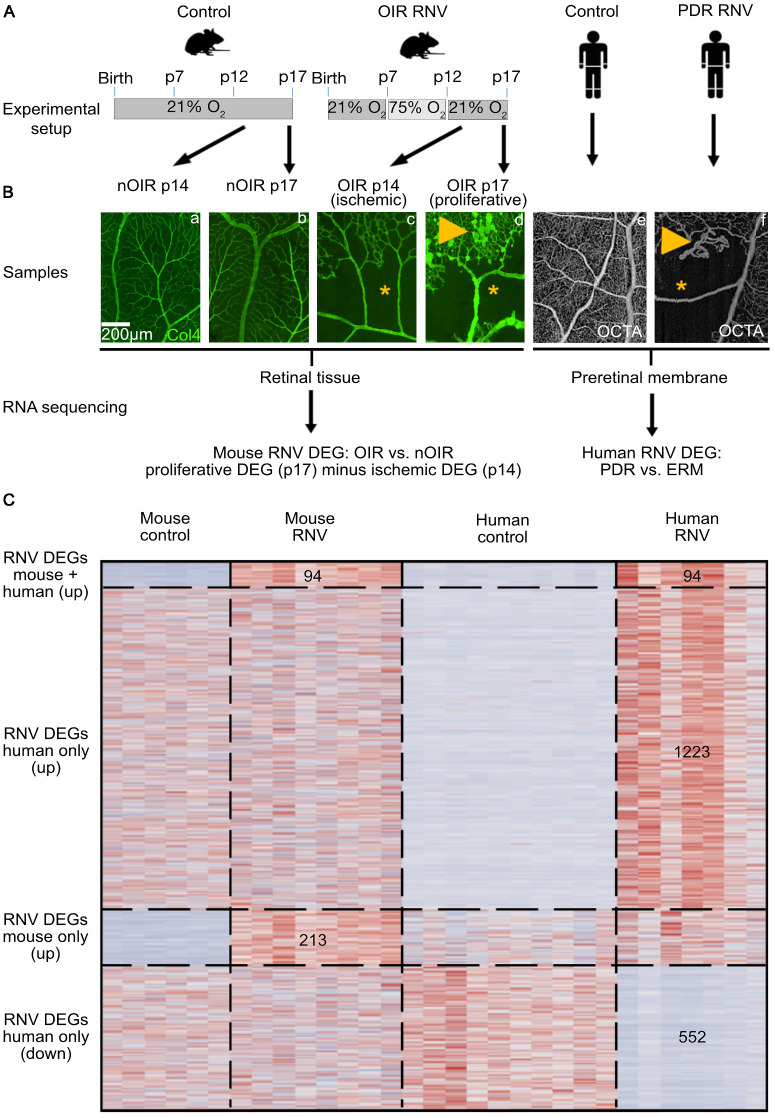
Experimental design of the study. For the murine part of the study, newborn pups constantly kept under normoxic conditions were used as controls and sacrificed at postnatal day (p) 14 or p17, respectively (*nOIR p14* and *nOIR p17*) (**A**). Physiological retinal vascularization was confirmed by Collagen IV staining of one eye of each litter (**Ba, Bb**). In the OIR group, newborn pups were brought up under normoxic conditions until p7 and were then transferred to a hyperoxic environment (75% oxygen). OIR pups were returned to room air on p12 and sacrificed on either p14 (*OIR p14*) or p17 (*OIR p17*). OIR retinas showed extensive avascular areas (*) on p14 (**Bc**) followed by retinal neovascularization (*arrowhead*) on p17 (**Bd**). In humans, retinal neovascularization membranes (RNV) extracted during routine vitrectomy from patients with proliferative diabetic retinopathy (PDR) were compared to control samples of (nondiabetic) epiretinal membranes (ERM), as described in a previous study.^24^ Similarly to our observations in mice, optical coherence tomography angiography (OCTA) of a patient with PDR (**Bf**) revealed extensive avascular areas (*), as well as consecutive development of RNV (*arrowhead*), whereas control patients showed intact retinal perfusion (**Be**). After RNA sequencing, bioinformatic analysis revealed DEG between PDR/OIR samples and their respective controls (**C**). Because whole retinal flatmounts were analyzed for murine experiments, whereas only preretinal membranes were collected as human samples, the DEG in OIR p14 retinas were deducted from those in OIR p17 to compensate for the effect of the ischemic retina. Finally, human and murine DEG were compared as shown on the heatmap revealing 94 DEG that were upregulated in both human and murine RNV, 1223 DEG that were upregulated in human RNV only, 213 DEG that were upregulated in mice only and 552 DEG that were downregulated in human RNV only (**C**).

Mice were exposed to the OIR model as described previously.^18^^,^^21^^,^^22^ Briefly, newborn pups (male and female) were kept under normoxic conditions until postnatal day (p) 7 and then transferred to a hyperoxic environment (75% oxygen). After transfer back to normoxic conditions on p12, they were sacrificed on p14 or p17 by cervical dislocation (Fig. 1A). These timepoints were chosen because in the OIR model at p14 the retina shows large ischemic areas without significant neovascularization, whereas at p17 the greatest extent of RNV is observed (Fig. 1B).^18^ Newborn mice that were constantly kept under normoxic conditions until p14 or p17 served as controls (nOIR p14 and nOIR p17, Fig. 1A). All mice were raised and kept at a constant temperature with a 12-hour light/dark cycle.

As previously described, only pups with normal weight gain up to p17 show a complete OIR phenotype with fully developed RNV.^19^ Pups undergoing the OIR experiments with a weight <5 g or >7.5 g at p17 were therefore excluded from analysis in our study. Overall, six mice from four litters were included in the OIR p14 group, eight mice from three litters in the OIR p17 group, seven mice from three litters in the nOIR p14 group, and six mice from two litters in the nOIR p17 group (Supplementary Table S1).

### Immunohistochemistry

To ensure efficient induction of the vascular phenotype in OIR eyes, the extent of RNV in the retina of one randomly selected pup of each litter was assessed by immunohistochemistry. After intracardiac perfusion with 4°C PBS, the eye was fixed in 4% paraformaldehyde for one hour at 4°C and processed for retinal flatmounting as described previously.^23^ A primary antibody against Collagen IV (COL4, goat anti-mouse, AB769; Merck Millipore, Billerica, MA, USA) was added at a dilution of 1:500 for two nights at 4°C on a shaker . A secondary antibody (Alexa Flour 488, donkey anti-goat, A11055; Life Technologies, Carlsbad, CA, USA) was added at a dilution of 1:500 overnight at 4°C on a shaker. Images of entire retinal flatmounts were taken using a Hamamatsu NanoZoomer S60 (Hamamatsu Photonics, Herrsching, Germany) to assess the development of ischemia and consecutive retinal neovascularization.^16^

### Total RNA Extraction

The fellow eye of the above-mentioned mice, as well as both eyes per animal pooled for other pups, were processed for retinal extraction straight after intracardiac PBS perfusion and enucleation. They were stored in RNAlater buffer (Qiagen, Hilden, Germany) for up to six days and shipped at 2° to 8°C to the RNA sequencing provider. Samples containing one or two retinas showed similar quality and quantity of extracted RNA. Total RNA extraction from murine retinas stabilized in RNAlater buffer was performed by the Genomics Core Facility “KFB–Center of Excellence for Fluorescent Bioanalytics” (University of Regensburg, Regensburg, Germany; www.kfb-regensburg.de) according to the “Purification of Total RNA From Animal and Human Tissue” protocol of the RNeasy Micro Kit (Qiagen). In brief, after centrifugation for five minutes at 5000*g* and removal of RNAlater, the tissue was disrupted and homogenized in 350 µL RLT buffer containing 1% beta-mercaptoethanol with Precellys CK14 ceramic beads (one cycle of 15 seconds at 5500 rpm) using a Precellys 24 homogenizer (Bertin Corp., Rockville, MD, USA). Next, samples were spun in a centrifuge for two minutes at full speed, and 350 µL of the cleared supernatant was transferred to a new tube. One volume of 70% ethanol was added, and the samples were applied to an RNeasy MinElute spin column followed by an on-column DNase digestion and several wash steps. Finally, total RNA was eluted in 14 µL of nuclease-free water. RNA purity and integrity were assessed on the Agilent 2100 Bioanalyzer with the RNA 6000 Nano LabChip reagent set (Agilent, Palo Alto, CA, USA).

### RNA Sequencing

The SMARTer Ultra Low Input RNA Kit for Sequencing v4 (Clontech Laboratories, Inc., Mountain View, CA, USA) was used to generate first-strand cDNA from approximately 1 ng of total RNA. Double-stranded cDNA was amplified by long-distance PCR (11 cycles) and purified via magnetic bead clean-up. Library preparation was carried out as described in the Illumina Nextera XT Sample Preparation Guide (Illumina, Inc., San Diego, CA, USA). Input cDNA 150 pg was tagmented (tagged and fragmented) by the Nextera XT transposome. The products were purified and amplified via a limited-cycle PCR program to generate multiplexed sequencing libraries. For the PCR step, 1:5 dilution of the unique dual indexing (i7 and I5) adapters was used. Libraries were quantified using the KAPA Library Quantification Kit–Illumina/ABI Prism User Guide (Roche Sequencing Solutions, Inc., Pleasanton, CA, USA). Equimolar amounts of each library were sequenced on an Illumina NextSeq 2000 instrument controlled by the NextSeq 2000 Control Software (NCS) v1.2.0.36376 using one 50 cycles P3 Flow Cell with the dual index, single-read run parameters. Image analysis and base calling were performed using the Real Time Analysis Software v3.7.17. The resulting .cbcl files were converted into .fastq files with the bcl2fastq v2.20 software.

### Human Samples

The RNA sequencing data of murine OIR retinas was combined with data of human RNV extracted during therapeutic pars plana vitrectomy from patients with PDR. Epiretinal membranes collected during vitrectomy for macular pucker served as controls (see Figs. 1Be, 1Bf). Ethics approval for this study was granted by the local Ethics Committee (University of Freiburg Medical Centre Ethics Committee Application No. 17/17) and written informed consent was obtained from each patient before tissue acquisition. RNA sequencing on these samples was performed as part of a previous project already published.^24^ Baseline characteristics of these patients are shown in Supplementary Table S1 and in more detail in the initial publication.

### Bioinformatics

Sequencing data were analyzed on the Galaxy web platform (usegalaxy.eu)^25^ as previously described.^26^ Quality control was performed with FastQC (Galaxy Version 0.73, http://www.bioinformatics.babraham.ac.uk/projects/fastqc/, last access on January 22, 2022). Reads were mapped to the human or mouse reference genome (human: Gencode release 35, mouse: Gencode M28) with RNA STAR (Galaxy Version 2.7.8a, default parameters).^27^ For each sample, the BAM files from each lane were combined in one BAM file per sample using Merge BAM files (Galaxy Version 1.2.0). Reads mapped to the human or mouse reference genome were counted by featureCounts (Galaxy Version 2.0.1, default parameters).^28^ The output of featureCounts was imported to RStudio (version 1.4.1103, R Version 4.0.3). Gene symbols and gene types were determined based on Ensembl (Release 105, download: 28.01.2022).^29^ Genes with zero reads in all samples were removed from further analysis. Principal component analysis (PCA)^30^ was applied to check for potential batch effect and outliers. One mouse (S4 from nOIR p14) was identified as an outlier sample because of high contamination with lens-specific genes (*Cryaa* and *Cryba1*) and excluded from further analysis. Normalized reads and differential gene expression were calculated using the Rpackage DESeq2 (version 1.34) with default parameters (Benjamini-Hochberg adjusted p-values^30^). Transcripts with log2 fold change (log2FC) >2 or <−2 and adjusted *P* value < 0.05 were considered as differentially expressed genes (DEG). Heatmaps were created with the R package ComplexHeatmap (version 2.1.0).^31^ Gene enrichment analysis and its visualization were performed using the R package clusterProfiler (version 4.2.2).^32^ Gene ontology (GO) analysis for clusters related to biological processes or molecular functions was performed based on the upregulated genes in RNV using the R function enrichGO of the clusterProfiler package with default parameters. Genes associated with the six most disease-relevant biological processes or molecular functions were illustrated using the R function cnetplot of the clusterProfiler package with default parameters. To investigate the enrichment of the VEGF pathway in human and murine RNV, we ran gene set enrichment analyses using the clusterProfiler R package based on the VEGF signaling pathway gene set (76 genes, entry number hsa04370) from the Kyoto Encyclopedia of Genes and Genomes database (download gene set on October 15, 2023, from https://www.gsea-msigdb.org/gsea/msigdb/cards/kegg_vegf_signaling_pathway). A ranked gene list was created for human PDR, OIR p14, and OIR p17, each ordered by the log2FC between disease and control. Other data visualization was performed using the ggplot2 R package.^33^

### Identification of Phylogenetically Conserved DEG

Although murine samples consisted of retinal flatmounts, human samples acquired during vitrectomy contained RNV/epiretinal membranes only. To account for the difference between these samples, DEG identified in OIR p14 mice when compared to their respective controls (nOIR p14) were deducted from the DEG in OIR p17 mice when compared to their respective controls (nOIR p17) to “subtract” the impact of the nonproliferative ischemic retina at OIR p14 from the proliferative and ischemic changes that are observed on OIR p17. This allowed the identification of DEG that were solely upregulated because of RNV in mice (RNV DEG, Fig. 1C). These RNV DEG were used for all comparisons with DEG from human samples. We also used normal retinal tissue as a second control group, that was obtained from patients undergoing enucleation due to choroidal melanoma at our institution. RNA sequencing on these samples was performed as part of a previous project already published.^34^

## Results

### Transcriptional Characterization of Murine RNV in the OIR Model

**Figure 2.**
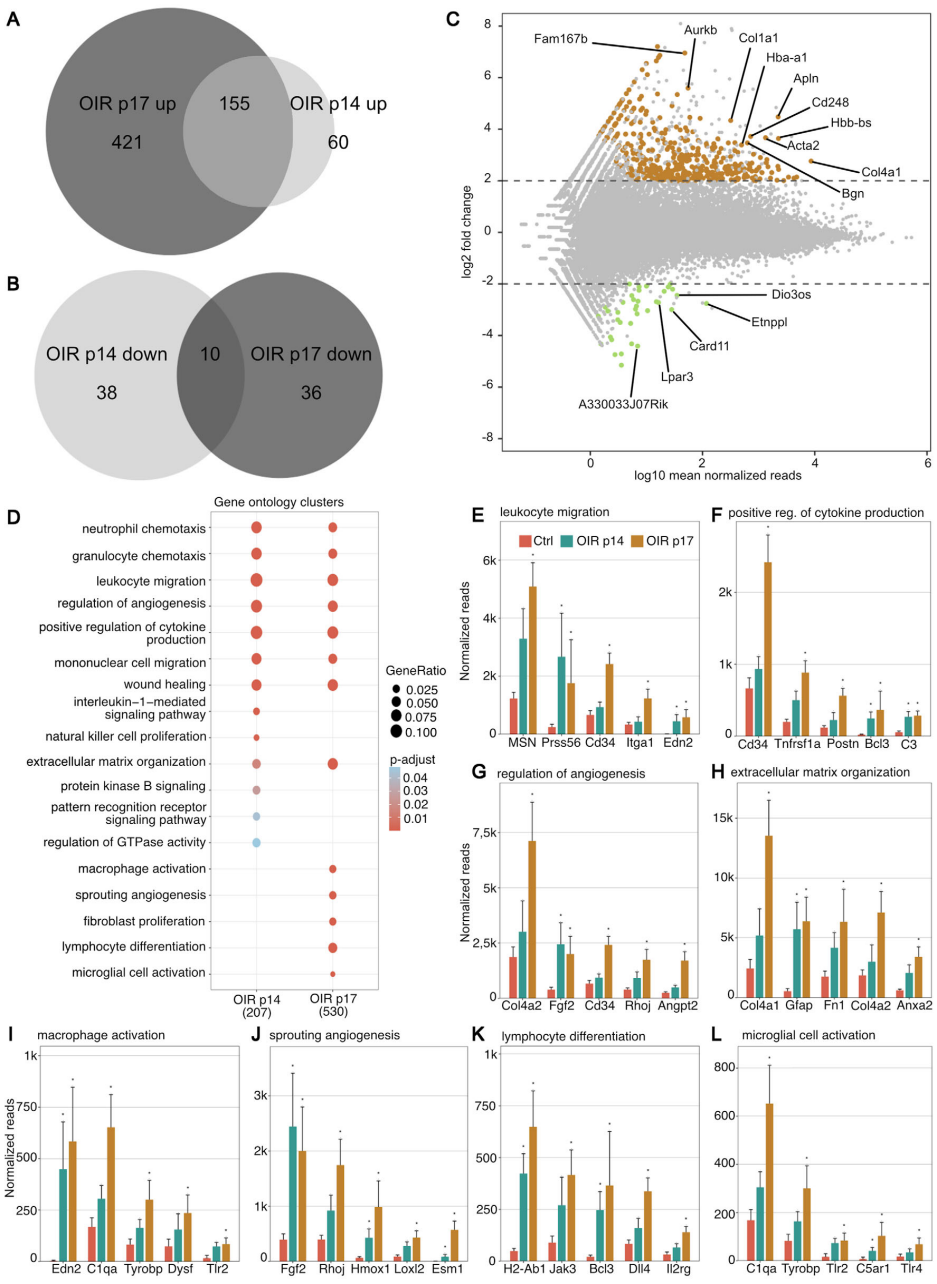
Gene expression and gene ontology clusters differentially expressed in the oxygen-induced retinopathy model. Venn diagrams visualizing DEG between OIR p14 and OIR p17 groups that were upregulated (**A**) or downregulated (**B**) compared to respective control samples. Genes with log2FC >2 or <−2 and adjusted *P* value <0.05 were considered as DEG. This showed on overlap of 155 upregulated and 10 downregulated DEG in both groups. (**C**) Scatter plot showing the logarithm of normalized mean reads versus log2 fold change at OIR p17. Upregulated DEG are shown in *brown*, whereas downregulated ones are shown in *green*. The 10 most highly expressed and upregulated, as well as the five most highly expressed and downregulated, DEG are annotated. (**D**) GO enrichment analysis based on the 207 DEG at p14 and 530 DEG at p17 that were included in the GO database. The adjusted *P* value of each GO term is color-coded. The gene ratio describes the ratio of the count to the number of all DEG and is coded by circle size. If no circle is shown at a time point, the adjusted *P* value was not statistically significant (>0.05). (**E–L**) The five most highly upregulated DEG within each GO cluster and their normalized reads in controls (nOIR p14 + nOIR p17, *red*), OIR p14 (*green*), and OIR p17 (*brown*) are shown.

Similar to the human situation in neovascular retinal diseases, the OIR model is characterized by a phase of retinal hypoxia (around p14), followed by a phase of RNV (around p17).^15^ To investigate the phase of retinal hypoxia in the OIR model, we first compared the transcriptional profile of the retina of mice at p14 with age-matched control mice (nOIR). We found that 263 genes were differentially expressed at p14 in the OIR group compared with controls (nOIR p14), with 82% (215) of those being upregulated and 18% (48) being downregulated (Figs. 2A, B). *Ccl4* (C-C Motif Chemokine Ligand 4), *Edn2* (Endothelin 2), *Timp1* (TIMP Metallopeptidase Inhibitor 1), and *Gfap* (Glial Fibrillary Acidic Protein) appeared among the most significantly upregulated genes at p14, whereas genes such as *Atp13a5* (ATPase 13A5) and *Gkn3* (Gastrokine 3) were among the most downregulated genes at p14 (Supplementary Fig. S1).

For OIR p17, 622 DEG were identified compared to respective controls (nOIR p17), 93% (576) of which were upregulated, whereas 7% (46) were downregulated (Figs. 2A, 2B). The genes that were most significantly upregulated on p17 included various types of collagens (*Col1a1*, *Col4a1*), as well as *Apln* (Apelin), *Acta2* (Actin Alpha 2, Smooth Muscle), and *Aurkb* (Aurora Kinase B) amongst others (Fig. 2C), whereas *Card11* (Caspase Recruitment Domain Family Member 11), *Etnppl* (Ethanolamine-Phosphate Phospho-Lyase), and *A33033J07Rik* belonged to the most significantly downregulated genes (Fig. 2C).

GO clusters that were upregulated on p14 and p17 included GO terms related to immunological processes (including neutrophil and granulocyte chemotaxis, leucocyte and mononuclear cell migration, and wound healing), as well as regulation of angiogenesis. In line with the typical OIR phenotype showing angiogenesis at p17 only but not yet at p14, genes associated with processes such as sprouting angiogenesis or fibroblast proliferation were only upregulated on p17, whereas the GO clusters interleukin-1-mediated signaling pathway and natural killer cell proliferation were only upregulated at p14 (Fig. 2D). For almost all upregulated GO clusters, DEG were more highly upregulated at p17 than at p14 (Figs. 2E–L).

### Comparison of Transcriptional Profiles of Human and Murine RNV

**Figure 3.**
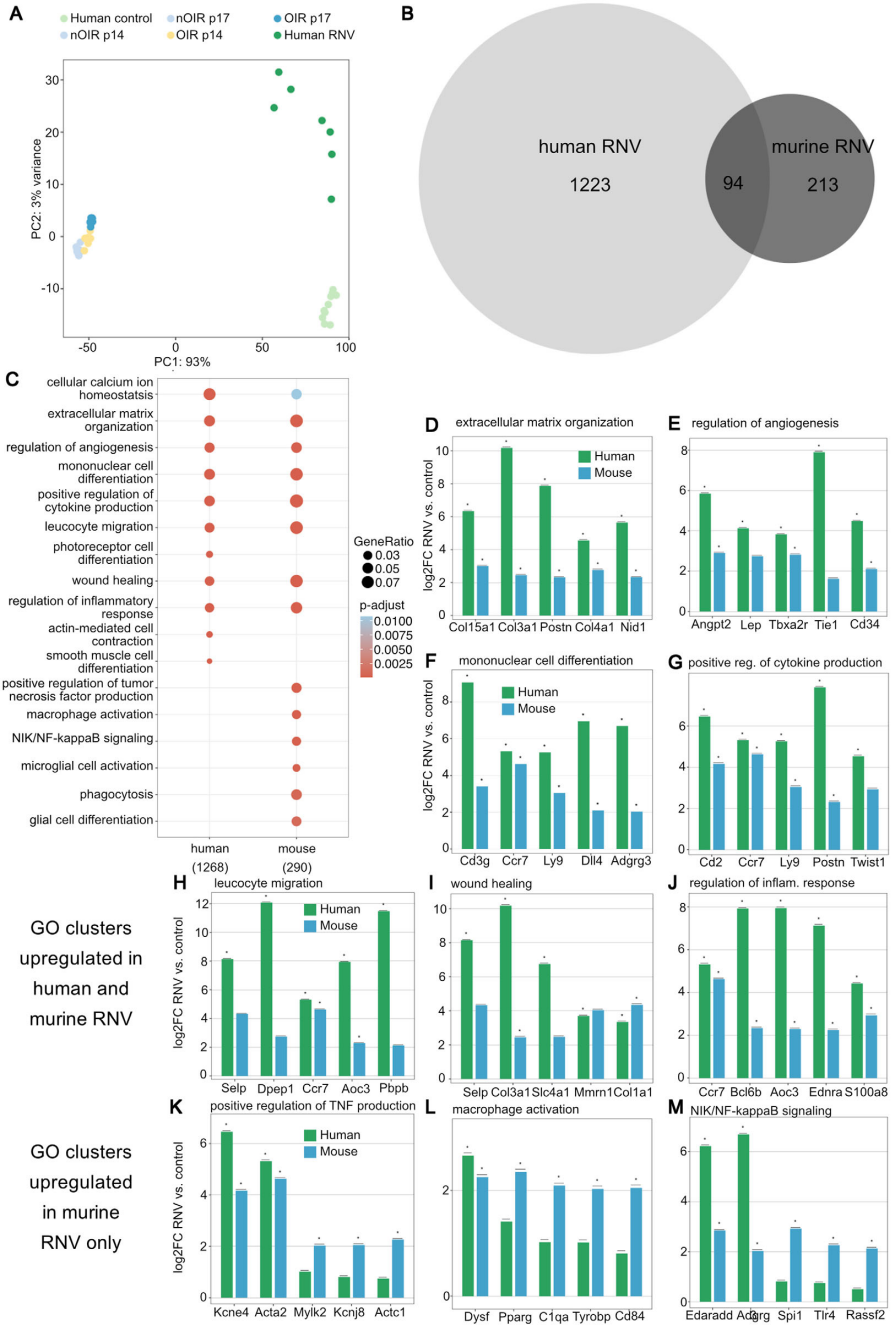
Comparison of transcriptome changes in human and murine retinal neovascularization. (**A**) PCA of human epiretinal gliosis membranes from macular pucker patients (*light green*), human RNV membranes from PDR patients (*dark green*), murine controls (nOIR p14 and nOIR p17, *light blue*), OIR p14 (*yellow*), and OIR p17 (*dark blue*) samples. A clear clustering of the samples from each group can be observed. (**B**) Venn diagram showing DEG that are upregulated in human and murine RNV, as well as those upregulated in both species. (**C**) GO terms upregulated in human and murine RNV reveal that genes involved in angiogenesis as well as immune response are upregulated in both species. The analysis is based on the 1268 human DEG and 290 murine DEG that were included in the GO database. The adjusted *P* value of each GO term is color-coded. The gene ratio describes the ratio of the count to the number of all DEG and is coded by circle size. (**D–J**) The five most highly upregulated DEG within each GO cluster and their logarithmic fold change in RNV versus control for human (*green*) and murine (*blue*) samples are shown. Almost all of these genes are more significantly upregulated in human than in murine samples. (**K–M**) The five most highly upregulated DEG in those pathways that are predominantly activated in murine RNV are presented with their logarithmic fold change in RNV versus control.

To identify the effect of retinal ischemia and hypoxia on the murine DEG and factors that are associated with retinal neovascularization, we next deducted the DEG of OIR p14 when compared to nOIR p14 (DEG of “ischemic retina”) from those of OIR p17 samples when compared to nOIR p17 samples (RNV DEG) and matched these RNV DEG to the human RNV data collected previously.^24^ PCA revealed differences between human and murine samples and further indicated a much higher inter-sample heterogeneity in human RNV in PDR compared to OIR retinas (Fig. 3A). Murine OIR samples and human PDR samples did, however, cluster in the same direction while the same was true for their respective controls, highlighting that similar transcriptomic changes can be observed in both species. In human samples, 1316 DEG were upregulated compared to their controls, only 7% (94) of which were also upregulated in the murine RNV DEG (Fig. 3B). With regard to downregulated factors, 566 DEG were found in the human membranes, less than 1% (n = 3) of which were also downregulated in murine RNV DEG. Thus only a small proportion of DEG were phylogenetically conserved between the OIR mouse model and human PDR, indicating that the model only partially reflects human pathology.

The GO clusters most significantly upregulated in human RNV included extracellular matrix organization, regulation of angiogenesis, leucocyte migration, wound healing, and regulation of inflammatory response, as previously published.^24^ These GO terms were also upregulated in the OIR model (Fig. 3C). We also investigated the role of the VEGF pathway in human and murine RNV, revealing an enrichment of the pathway in both species, with increasing enrichment scores from OIR p14 (0.302) to OIR p17 (0.411), corresponding to the disease phenotype seen in the OIR model. Similarly, the VEGF pathway was enriched in human PDR membranes (enrichment score = 0.472). The GO terms photoreceptor cell differentiation, actin-mediated cell contraction, and smooth muscle cell differentiation were only upregulated in human, but not in murine samples. Positive regulation of tumor necrosis factor production, NIK/NF-kappaB signaling, microglial cell activation, phagocytosis, and glial cell differentiation, by contrast, were upregulated in murine RNV only (Fig. 3C). Focusing on the top five DEG of each GO cluster enriched in both species, most DEG were more highly upregulated in human than in murine RNV (Figs. 3D–J). For those GO terms that only showed an overall upregulation in murine RNV and not in human RNV, individual genes were nevertheless also upregulated in human RNV, among them *KCNE4* (potassium voltage-gated channel subfamily E regulatory subunit 4), *ACTA2* (actin alpha 2, smooth muscle), *DYSF* (dysferlin), *EDARADD* (EDAR-associated death domain), or *ADGRG3* (adhesion G protein-coupled receptor G3), whereas others, such as *RASSF2* (Ras association domain family member 2), were not (Figs. 3K–N). Our results indicate that the molecular overlap between murine and human RNV is relatively small, although similar biological processes are enriched in both species. These findings demonstrate the potential and limitations of OIR experiments as a model for human PDR.

### Identification of Phylogenetically Conserved Mediators Between OIR and PDR Samples

**Figure 4.**
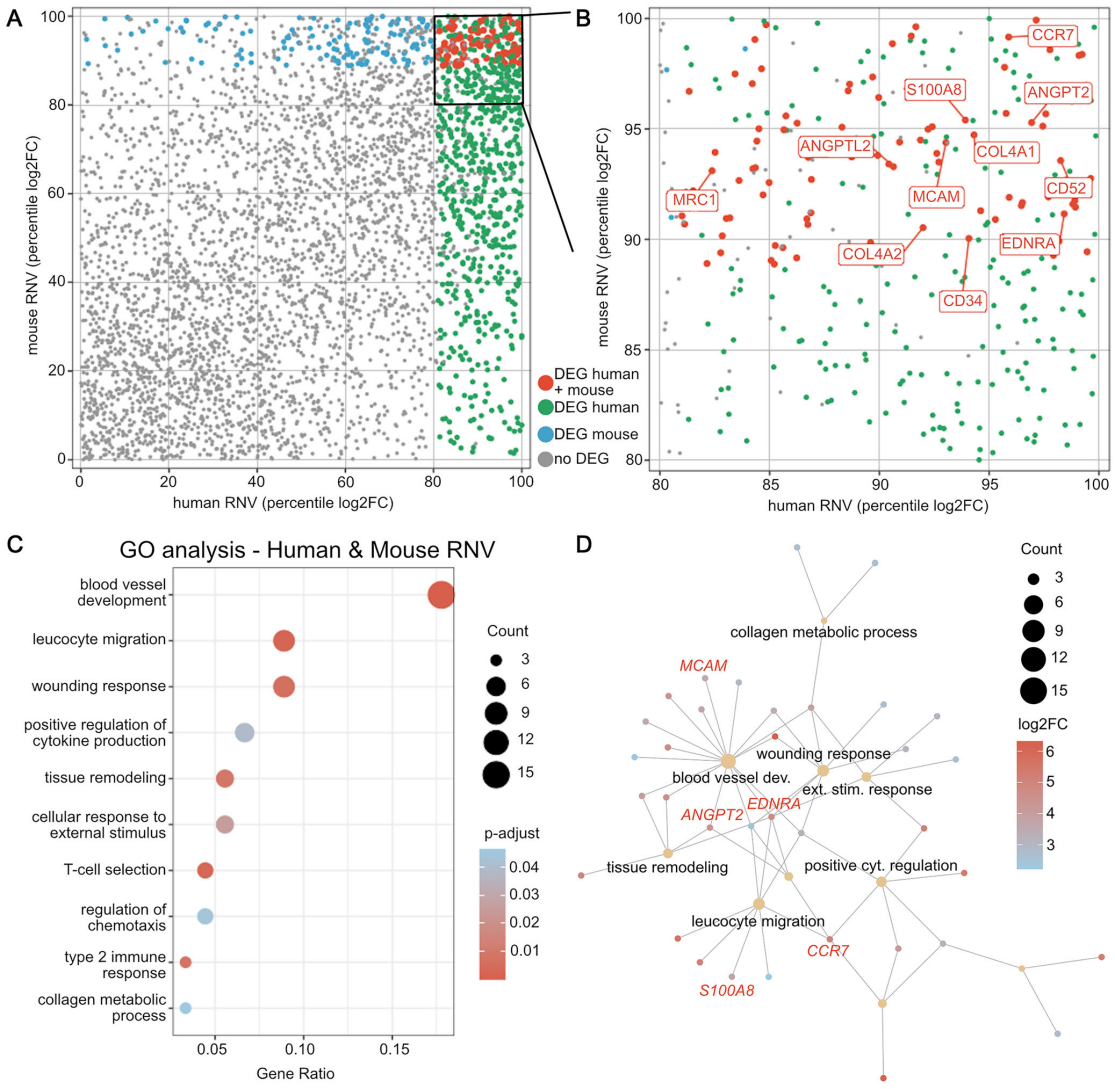
Identification of phylogenetically conserved differentially expressed genes in human and murine retinal neovascularization. (**A**) Overview of RNV according to their percentile logarithm twofold change in human versus mouse RNV. Genes shown in *red* represent DEG in both species, those in *green* are DEG in human samples only and those in blue are DEG in murine samples only. Gray denotes that the gene was not differentially expressed in either species. (**B**) The overlapping DEG above the eightieth percentile are shown in *red*, and some exemplary transcripts are labeled. (**C**) GO clusters that contained the most phylogenetically conserved DEG included blood vessel development, leucocyte migration, and response to wounding. Hereby, circle size encodes the number of associated upregulated DEG, whereas the adjusted *P* values are color coded. The gene ratio on the x axis describes the ratio of the DEG count within each cluster to the number of all phylogenetically conserved DEG. (**D**) Network analysis of GO terms and some associated individual genes that were most significantly upregulated. *Ochre circle* size codes for the number of upregulated DEG in each GO cluster, whereas the color of each gene represents its log2-fold change. Selected DEG mentioned in the main text are highlighted in *red*, while associated GO terms are labeled *black*. The full network analysis including all labels is shown in Supplementary Figure S3.

Comparing significantly upregulated genes in human and murine RNV (proliferative DEG at p17 minus ischemic DEG at p14, Fig. 1B) identified 94 phylogenetically conserved mediators of RNV development (Fig. 4A, Supplementary Table S2). These included various collagen types (*COL4A1*, *COL4A2*), cluster of differentiation (CD) molecules *CD34* and *CD52*, *ANGPT2* (angiopoetin 2), *ANGPTL2* (angiopoietin-like 2), *S100A8* (S100 calcium-binding protein A8), *MCAM* (melanoma cell adhesion molecule), *MRC1* (mannose receptor C-type 1), *EDNRA* (endothelin receptor type A), and *CCR7* (C-C motif chemokine receptor 7) (Fig. 4B). Most of these genes were not only upregulated compared to ERM membranes but also compared to healthy retinal tissue (Supplementary Fig. S2). Phylogenetically conserved DEG were mostly associated with the GO terms blood vessel development, leucocyte migration, response to wounding, and positive regulation of cytokine production (Fig. 4C). A network analysis of GO terms and associated DEG revealed that *ANGPT2, S100A8, EDNRA, MCAM,* and *CCR7* were key factors involved in these pathophysiologically relevant biological processes (Fig. 4D, in more detail in Supplementary Fig. S3). These conserved factors may represent potential treatment targets for proliferative retinopathies that could be validated in the OIR model and may thus be of special interest for future studies.

## Discussion

Even though PRP and anti-VEGF injections have improved outcomes in patients with RNV associated with ischemic retinopathies, it still remain a major cause of blindness worldwide.^35^ Previous studies report that about 30% of patients with PDR do not show a sufficient response to either treatment,^12^ which points to the urgent need for animal models to identify alternative treatment targets. Our study used comparative transcriptomics between murine and human RNV to study how closely the OIR mouse model reflects the changes observed in neovascular membranes in human PDR, and only a comparably small overlap was found. We nevertheless identified phylogenetically conserved factors of RNV formation such as *ANGPT2, S100A8, MCAM, EDNRA, MRC1,* and *CCR7* that warrant further investigation.

The OIR mouse model has been used extensively as a model to study RNV development in vivo.^16^^,^^18^^,^^19^ Because its retinal phenotype of extensive ischemia and subsequent development of neovascular tufts closely resembles the clinical phenotype of human ischemic and proliferative retinopathies (see Fig. 1B), it is frequently used as an animal model to study RNV in ROP, as well as PDR. Our study, however, shows only a comparatively small overlap between human and murine RNV DEG (compare Fig. 3B). Explanations for this discrepancy, other than species-specific factors, could be the age difference between newborn pups and human adults, but also metabolic differences between nondiabetic OIR mice and humans with an underlying metabolic disease that causes generalized microvascular damage.^36^ Although murine diabetes models currently available may mimic these generalized changes, none of these models develop RNV, which limits their application when studying PDR.^20^ Because sample clustering on PCA (Fig. 3A) and upregulated GO clusters (Fig. 3C) hint at a similar direction of transcriptomic changes in both species and due to the lack of other suitable RNV animal models, the OIR mouse model may still represent one of the most useful models to identify and validate new therapeutic targets for human proliferative retinopathies. Based on our results, the OIR model may be particularly useful when investigating pathways related to extracellular matrix organization, regulation of angiogenesis, mononuclear cell differentiation, positive regulation of cytokine production, leucocyte migration, wound healing, and regulation of inflammatory response. In contrast, processes such as tumor necrosis factor production, macrophage activation, NIK/NF-kappaB signaling, and microglial cell activation were less similar between human and mouse.

This seems especially plausible because animal models in general often only poorly reflect changes observed in human disease.^37^^,^^38^ When analyzing genomic responses observed in different animal models and respective human counterparts that they were supposed to mimic, Seok et al.^37^ found a close to random matching between species. Previous work by our group correlating transcriptional changes in human choroidal neovascularization (CNV) because of age-related macular degeneration with murine laser-induced CNV, found that only about 11% of DEG upregulated in humans were also upregulated in the mouse model.^38^ Nevertheless, our previous study identified fibroblast growth factor inducible-14 (FN14) as a novel phylogenetically conserved mediator of CNV, and its inhibition via intravitreal antibody treatment resulted in a significant reduction of CNV size in vivo. While animal models may not resemble the entire complexity of human pathology, they are still valuable to study specific aspects of human disease, as demonstrated in our present and previous studies on RNV in PDR and CNV in age-related macular degeneration. Most importantly, animal models are essential to model the complex environment within organisms and organs, such as the eye including its large diversity of cell types, which in vitro models still fail to accomplish. This complexity is critical to understanding human pathophysiology and establishing novel therapeutic targets.

Our current study not only assessed alterations in the murine transcriptome when RNV had already developed (OIR p17), but also changes seen at a preproliferative stage with widespread ischemia (OIR p14). Our data reveal that, even at a preproliferative stage, an extensive upregulation of factors that promote angiogenesis can be observed (Fig. 2). This suggests that an early inhibition of the release of these factors could mitigate proangiogenic stimuli, which may eventually yield better clinical outcomes. In line with this hypothesis, a large randomized controlled trial found a slightly lower incidence of severe visual loss in patients that received PRP for severe nonproliferative diabetic retinopathy (also referred to as “preproliferative” in the clinical context) than in patients who had already developed PDR.^5^ Novel lasers, which have been developed after that study was performed, may have an even more favorable side effect profile and may thus even strengthen the advantage of treating at the prepoliferative stage.^39^ Taken together, these data may suggest that treatment of the preproliferative rather than the proliferative disease stage may be beneficial in ischemic retinopathies. This advantage could be furthered by specifically targeting some of the factors we identified in this study early, thus avoiding side effects of PRP such as peripheral scotoma.

With regard to specific targets, one of the most upregulated and most strongly phylogenetically conserved genes in our study was *ANGPT2* (Angiopoietin 2). *ANGPT2* is already considered a major factor in choroidal and retinal vascular disease,^40^ and it has been shown that heterozygous *Anpgt2* knockout mice in the OIR model develop significantly less neovascularization.^41^ Recently, Faricimab, a bispecific antibody targeting the ANGPT2, as well as the VEGF pathway, has been introduced.^42^ In a phase 3 trial, it was shown to be effective for the treatment of diabetic macular edema and potentially even to allow for longer dosing intervals than injections that target VEGF only.^43^ It subsequently received FDA approval for diabetic macular edema, as well as for neovascular age-related macular degeneration.^44^ Our results could hint at a potential role for faricimab in the treatment of RNV in proliferative retinopathies, which, to our knowledge, has not been studied in large, randomized controlled trials so far.

In addition to *ANGPT2*, our study reveals a plethora of angiogenic and/or inflammatory factors such as *S100A8*, *MCAM*, *EDNRA*, and *CCR7*, that represent promising candidates for future clinical studies. *S100A8* (S100 calcium binding protein A8) is involved in inflammatory processes such as neutrophil chemotaxis and adhesion. It has been studied as part of immune response,^45^ but has also been investigated as a biomarker in myocardial infarction,^46^ breast cancer,^47^ as well as age-related macular degeneration.^48^ In a transcriptome analysis of peripheral blood neutrophils from patients with type 2 diabetes compared to healthy controls, *S100A8* was among the most significantly upregulated factors in diabetic patients.^49^ With regard to diabetes and the eye, systemic levels of the S100A8 and S100A9 protein strongly correlated with the severity of diabetic retinopathy in patients with type 2 diabetes.^50^ This indicates a role of *S100A8* in the pathogenesis of diabetic retinopathy and its possible usefulness as a prognostic biomarker. Its inhibition as a potential treatment of ischemic retinopathies, however, remains to be elucidated.

*MCAM* (melanoma cell adhesion molecule, also known as CD146) is involved in angiogenesis and inflammation and has been studied extensively.^51^ Even its role in diabetic retinopathy has already been investigated.^52^^,^^53^ In a study using the OIR model, its inhibition led to a reduction in formation of neovascularization via the VEGFR2 (vascular endothelial growth factor receptor 2) pathway, and it was considered a promising treatment target for PDR.^54^ Its effectiveness for the treatment of human RNV remains to be investigated.

*EDNRA* (endothelin receptor type A) is the receptor of endothelin 1, which is a long-acting vasoconstrictor and has already received a lot of attention in various ophthalmic diseases including PDR.^55^ For example, it was found to be more abundant in PDR membranes than in epiretinal gliosis membranes from macular pucker patients.^56^ *EDNRA* itself has been implicated in diabetic kidney disease, where a study found that certain polymorphisms in the *EDNRA* and *EDN1* gene acted protective against the development of the disease.^57^ Studies on the role of *EDNRA* in PDR, however, are still lacking.

*CCR7* (C-C Motif Chemokine Receptor 7), a receptor of cytokines CCL19 and CCL21, is implicated in the homing of T cells in lymph nodes,^58^ and it has mostly been studied with regard to its proangiogenic effect on cancer cells through an increase in VEGF levels.^59^ With regard to the eye, it has so far only been described in dry eye disease,^60^ but has not been linked to diabetic retinopathy yet. These genes may thus all represent promising treatment targets for proliferative retinal diseases and warrant further investigation.

Our study has some limitations. Although whole retinal tissue was analyzed from the mouse, we investigated human preretinal RNV membranes, because the collection of whole retinal tissue from living PDR patients is not feasible and postmortem tissue may be limited by lower sample quality.^48^^,^^61^^,^^62^ We acknowledge that ERM tissue as a control may lead to underestimation of genes associated with processes such as cellular proliferation. To account for a potential influence of retinal tissue in the OIR mouse model, we assessed DEG of the ischemic retina (OIR vs. control at p14) and deducted these genes from the DEG of the ischemic retina with retinal neovascularization (OIR vs. control at p17). We acknowledge the possibility that some retinal genes were differentially expressed at p17, but not at p14. However, the murine RNV DEG were not involved in retina-specific pathways but were almost exclusively enriched in angiogenesis- and immune-related pathways, indicating that the few retina-specific DEG were of minor biological relevance. Our strategy represents the clinically and scientifically most feasible approach to compare human and murine RNV with the highest possible data quality. The primary goal of our study was to assess how closely the nondiabetic OIR model resembles human PDR and for which aspects of human pathophysiology the OIR model is suitable for future functional validation experiments. Importantly, we did not look at the protein level. Our data provides a valuable foundation for future studies to test the therapeutic efficacy of the identified mediators in the OIR model with the potential to translate into pharmacological targets in human clinical trials.

In conclusion, this study shows a significant upregulation of inflammatory and proangiogenic factors at both p14 and p17 in the OIR mouse model. Comparative and unbiased transcriptomics identified only a small overlap of DEG between murine and human RNV membranes, although similar biological processes were enriched in both settings. These findings demonstrate the potential and limitations of the OIR mouse as a model for human RNV in PDR. The interspecies molecular comparison identified 94 phylogenetically conserved mediators of RNV, including *ANGPT2, S100A8, MCAM, EDNRA,* and *CCR7*. These potential therapeutic targets could be validated in future studies using the OIR model, potentially leading to new or repurposed therapies for human proliferative retinal diseases.

## Supplementary Material

Supplement 1

Supplement 2

Supplement 3

Supplement 4
