# Supplementary material for: Transcriptional Comparison of Human and Murine Retinal Neovascularization
Source: Invest Ophthalmol Vis Sci. 2023 Dec 28;64(15):46. doi: 10.1167/iovs.64.15.46 (PMC10756240; doi:10.1167/iovs.64.15.46)
Supplement: Supplement 1 [file iovs-64-15-46_s001.pdf]

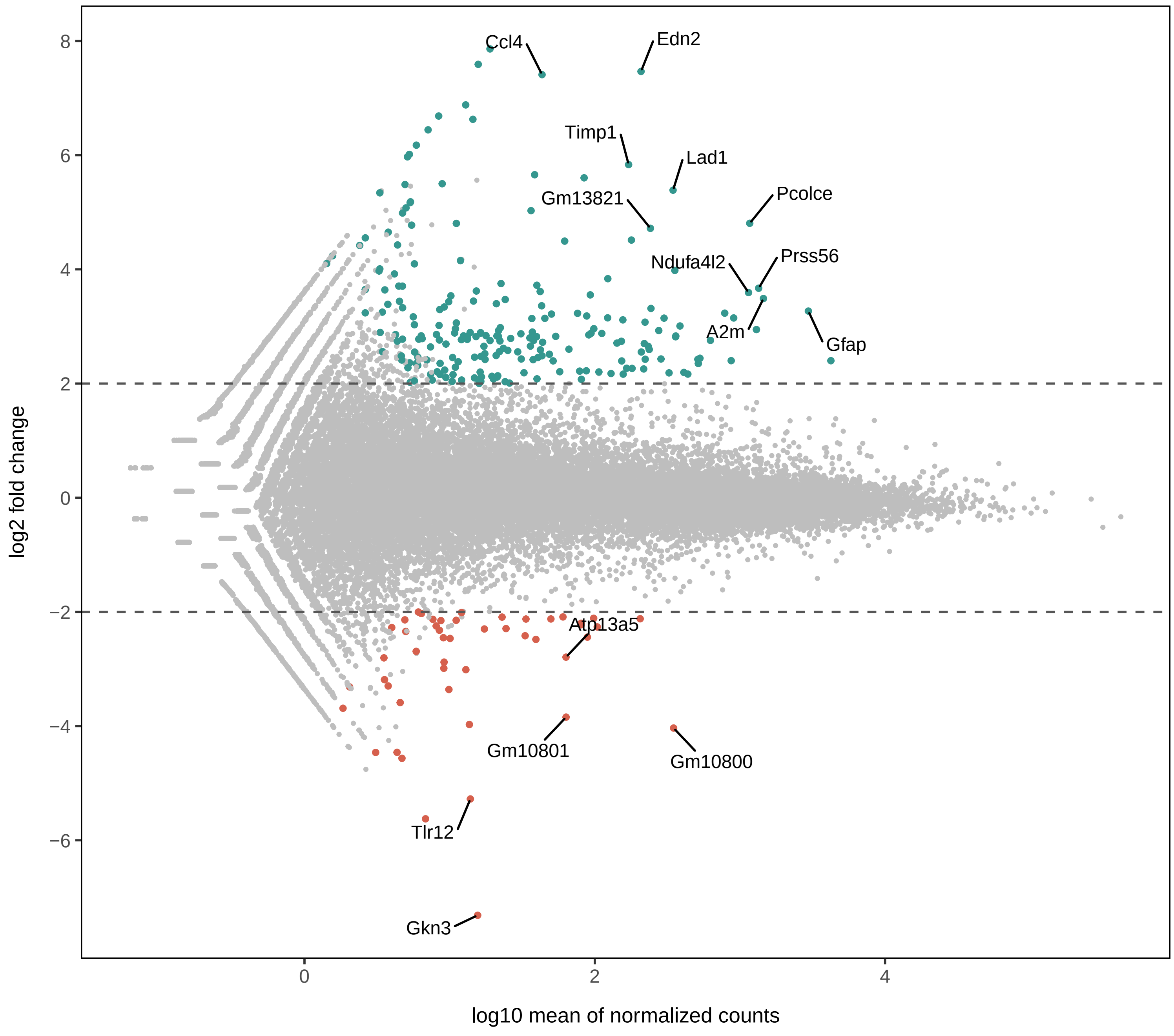

log2 fold change
log10 mean of normalized counts
8
6
4
2
0
−2
−4
−6
0
2
4
Ccl4
Edn2
Timp1
Lad1
Gm13821
Pcolce
Ndufa4l2
Prss56
A2m
Gfap
Atp13a5
Gm10801
Gm10800
Tlr12
Gkn3

Suppl. Figure 1: Differentially expressed genes at OIR p14. Scatter plot showing the logarithm of normalized mean reads versus log2 fold change at OIR p14. Upregulated DEG are shown in turquoise, while downregulated ones are shown in red. The 10 most highly expressed and upregulated as well as the 5 most highly expressed and downregulated DEG are annotated.
