# Supplementary material for: Transcriptional Comparison of Human and Murine Retinal Neovascularization
Source: Invest Ophthalmol Vis Sci. 2023 Dec 28;64(15):46. doi: 10.1167/iovs.64.15.46 (PMC10756240; doi:10.1167/iovs.64.15.46)
Supplement: Supplement 2 [file iovs-64-15-46_s002.pdf]

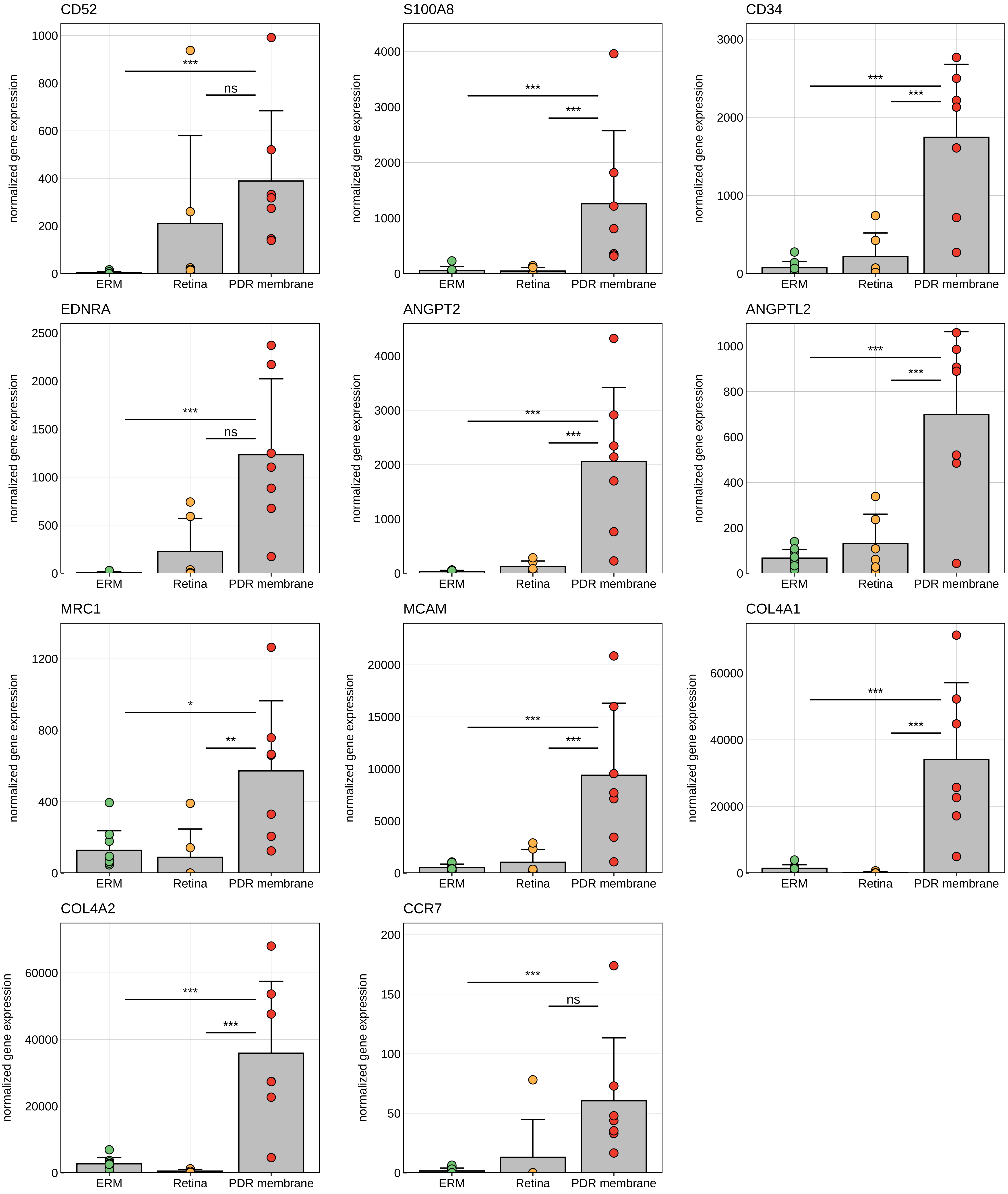
CD52
normalized gene expression
0
200
400
600
800
1000
***
ns
ERM
Retina
PDR membrane
S100A8
normalized gene expression
0
1000
2000
3000
4000
***
***
ERM
Retina
PDR membrane
CD34
normalized gene expression
0
1000
2000
3000
***
***
ERM
Retina
PDR membrane
EDNRA
normalized gene expression
0
500
1000
1500
2000
2500
***
ns
ERM
Retina
PDR membrane
ANGPT2
normalized gene expression
0
1000
2000
3000
4000
***
***
ERM
Retina
PDR membrane
ANGPTL2
normalized gene expression
0
200
400
600
800
1000
***
***
ERM
Retina
PDR membrane
MRC1
normalized gene expression
0
400
800
1200
*
**
ERM
Retina
PDR membrane
MCAM
normalized gene expression
0
5000
10000
15000
20000
***
***
ERM
Retina
PDR membrane
COL4A1
normalized gene expression
0
20000
40000
60000
***
***
ERM
Retina
PDR membrane
COL4A2
normalized gene expression
0
20000
40000
60000
***
***
ERM
Retina
PDR membrane
CCR7
normalized gene expression
0
50
100
150
200
***
ns
ERM
Retina
PDR membrane

Suppl. Figure 2: Phylogenetically conserved key mediators of RNV are not only upregulated compared to epiretinal membranes, but also compared to healthy retinal tissue. Bar graphs showing normalized gene expression. Each point is one sample.
