# Supplementary material for: Transcriptional Comparison of Human and Murine Retinal Neovascularization
Source: Invest Ophthalmol Vis Sci. 2023 Dec 28;64(15):46. doi: 10.1167/iovs.64.15.46 (PMC10756240; doi:10.1167/iovs.64.15.46)
Supplement: Supplement 3 [file iovs-64-15-46_s003.pdf]

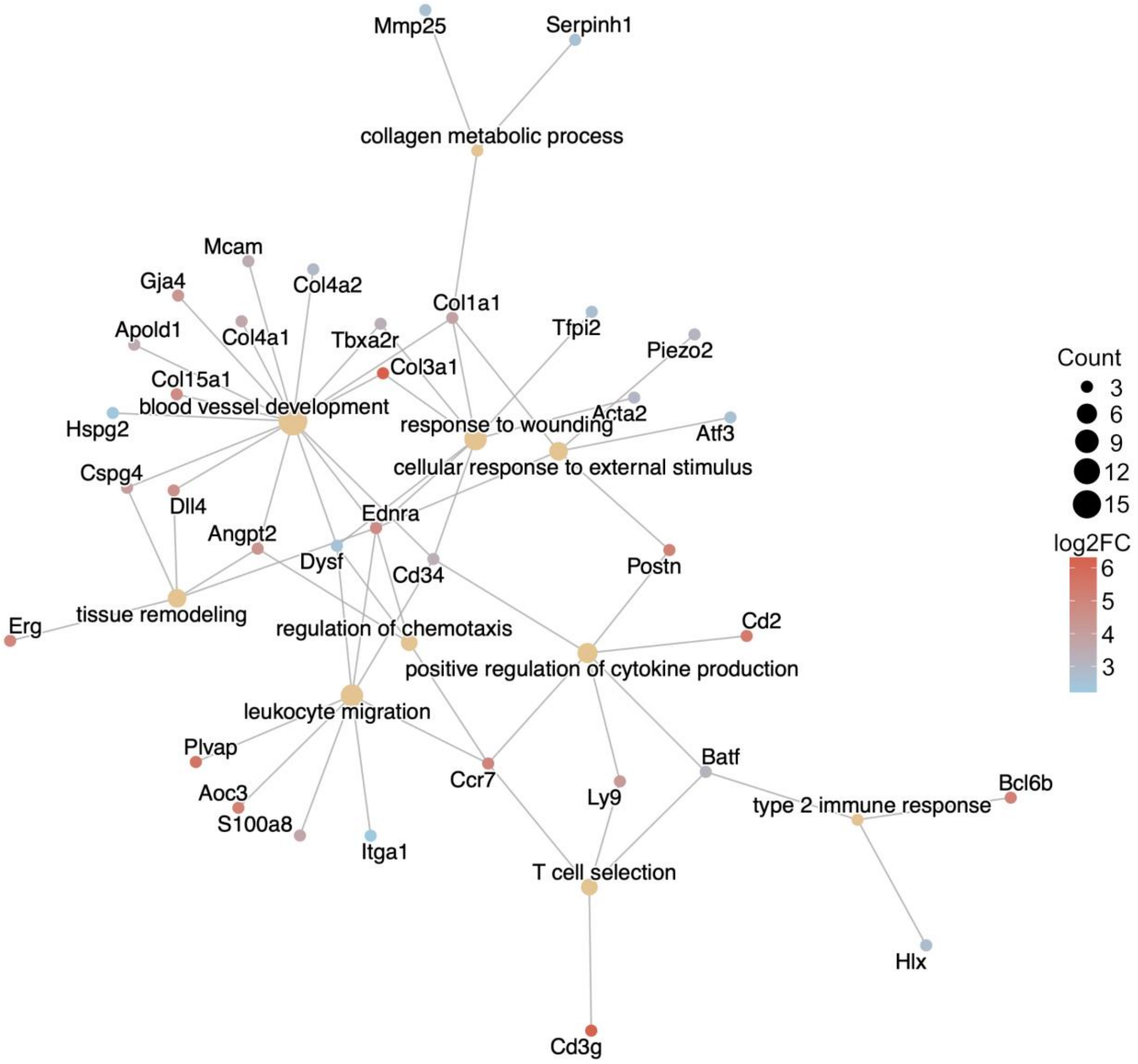

Suppl. Figure 3: Full network analysis of gene ontology (GO) clusters and associated differentially expressed genes (DEG) most significantly upregulated. Circle size (ocher circles) codes for the number of upregulated DEG in each GO cluster, while the color of each gene (other circles) represents the respective log2 fold change (log2FC).
