# Supplementary material for: Transcriptional Comparison of Human and Murine Retinal Neovascularization
Source: Invest Ophthalmol Vis Sci. 2023 Dec 28;64(15):46. doi: 10.1167/iovs.64.15.46 (PMC10756240; doi:10.1167/iovs.64.15.46)
Supplement: Supplement 4 [file iovs-64-15-46_s004.pdf]

	Murine				Human	
	nOIR p14	nOIR p17	OIR p14	OIR p17	ERM	PDR
**n**	7	6	6	8	10	7
**Number of litters**	3	2	4	3	-	-
**% female**	57%	33%	67%	25%	30%	29%
**mean weight (SD)**	7.0 (0.6)	7.1 (1.1)	4.7 (0.4)	5.8 (0.8)	-	-
**mean age (SD)**	14 days	17 days	14 days	17 days	71 years (6.8)	38 years (14.8)

Suppl. Table 1: Characteristics of the murine and human samples included in this study. Six or more mice from 2 or more litters were analyzed for each murine group. The percentage of female mice varied between p14 and p17 groups, but it was similar within the comparisons between controls (nOIR) and oxygen-induced retinopathy (OIR) groups at each timepoint, as well as between the epiretinal membrane (ERM) and proliferative diabetic retinopathy (PDR) groups. OIR pups were only included between the weight limits of 5 to 7.5 g for p17 in keeping with previous reports.[19]

Sym_human	Sym_mouse	log2FC_human	p_adj_human	log2FC_mouse	p_adj_mouse
COL3A1	Col3a1	10,17444631	2,80304E-53	2,450114092	0,009085394
COL5A2	Col5a2	2,978040826	1,39181E-14	2,576228784	1,98336E-08
KCNE4	Kcne4	5,317856859	1,84086E-18	2,34508251	0,005515108
ASPM	Aspm	2,147561217	0,01054472	2,626799608	0,01987514
LY9	Ly9	5,250162301	1,4084E-09	3,044952022	0,028400154
HLX	Hlx	2,500982796	1,15232E-08	2,991870314	1,14219E-08
CENPF	Cenpf	2,333585047	1,04425E-09	2,508081328	8,385E-08
ATF3	Atf3	2,649435215	6,96912E-07	2,564181064	6,61814E-05
CD34	Cd34	4,488775886	4,11571E-29	2,108797403	2,22801E-05
CCDC3	Ccdc3	2,197601827	7,46426E-05	2,215114952	0,002247177
MRC1	Mrc1	2,139150896	0,011776623	2,49236979	0,034683967
PKN3	Pkn3	2,643562489	1,92112E-13	2,213321263	4,83301E-07
ANGPTL2	Angptl2	3,357434718	4,64825E-22	2,51553891	7,5937E-10
OLFML2A	Olfml2a	2,020790437	5,34888E-09	2,368022716	2,95077E-09
SLC43A1	Slc43a1	2,328438834	9,36028E-09	4,447201144	1,50646E-09
DLL4	Dll4	6,947336197	7,37297E-35	2,096677036	0,006833169
DUSP2	Dusp2	3,14929292	2,26236E-10	2,094003739	0,001041483
MALL	Mall	3,849685199	1,28449E-09	2,844788857	0,000341947
CD93	Cd93	3,331205238	5,74041E-39	2,527794122	6,16107E-18
REM1	Rem1	3,680313729	7,38617E-08	2,716837112	0,003109857
TPX2	Tpx2	2,024209388	4,84747E-07	2,372627568	7,03351E-07
FAM83D	Fam83d	3,163964154	6,95194E-06	3,497689535	0,000112072
POSTN	Postn	7,86740624	1,41815E-33	2,316130764	0,012923191
RARRES1	Rarres1	2,451516236	1,50481E-06	2,079471299	0,002735629
FCRL2	Fcrls	2,587971955	0,00030853	2,884663561	2,60485E-09
S100A8	S100a8	4,419544302	3,24588E-08	2,937755462	0,046819248

CD2	Cd2	6,457310412	5,49318E-10	4,162155054	0,029778615
COL15A1	Col15a1	6,33910222	1,29492E-44	3,00650078	2,06572E-07
RHBDL2	Rhbdl2	2,060703357	1,39086E-06	2,387259063	7,15605E-06
GJA4	Gja4	6,502901629	1,69598E-20	2,112689175	0,043366886
FAM167B	Fam167b	5,98893846	9,4518E-42	6,953455593	6,1375E-15
CD52	Cd52	7,037177864	6,65946E-30	2,548094231	0,001840974
HSPG2	Hspg2	2,24171976	3,77152E-11	2,443340508	4,26236E-10
FBLIM1	Fblim1	3,429373062	3,18305E-39	2,705216598	1,06667E-17
MSX1	Msx1	4,976204944	4,22468E-15	2,212062941	0,015978524
NCAPG	Ncapg	2,478865456	7,54491E-06	2,075141519	0,005593165
TFPI2	Tfpi2	2,044791589	0,007415082	3,291383995	0,000775609
GNG11	Gng11	2,203345441	1,51228E-18	2,215749084	1,54344E-14
PRR15	Prr15	2,000822368	0,001171001	2,223546816	0,013544865
DYSF	Dysf	2,659077142	7,88033E-14	2,253569732	1,57946E-07
EGR4	Egr4	2,348256558	0,047771437	2,819206698	9,24126E-05
CLEC1A	Clec1a	3,213504573	3,9413E-13	2,583212485	1,4021E-06
APOLD1	Apold1	4,715142217	5,54435E-25	2,268408688	8,62462E-05
ARHGDIB	Arhgdib	2,16986336	3,33135E-22	2,046715037	4,13021E-15
FOSB	Fosb	4,262063422	1,09925E-07	3,289978419	0,002292114
CKM	Ckm	3,961828621	3,45964E-06	2,5946809	0,009089245
EXOC3L2	Exoc3l2	9,151456676	4,42868E-52	2,360438786	0,000943848
SERPINH1	Serpinh1	2,665845532	4,05659E-42	2,447813223	2,21098E-28
IL21R	Il21r	2,171522969	0,000948169	2,114296235	0,026630619
COL4A1	Col4a1	4,556990327	2,07229E-32	2,766184292	1,01285E-09
COL4A2	Col4a2	3,710794894	9,51364E-25	2,14746048	8,16667E-07
ANGPT2	Angpt2	5,843000169	2,83034E-62	2,903063655	1,36215E-12
PLVAP	Plvap	9,116611169	9,76184E-55	2,049652341	0,013945581

TPM4	Tpm4	2,117732946	5,4246E-22	2,010086738	9,32227E-16
EDNRA	Ednra	7,127533046	3,02704E-40	2,247872422	0,001519476
ADGRG3	Adgrg3	6,688700741	1,47587E-13	2,031559328	0,023772328
CNN1	Cnn1	2,360129952	1,11346E-06	2,359431632	0,001089498
FLI1	Fli1	2,450035551	6,30951E-23	2,007784194	5,72079E-12
MCAM	Mcam	4,123700499	5,37079E-34	2,702767555	9,60306E-12
CD3G	Cd3g	9,063047643	3,44548E-23	3,399972943	0,029625339
CSPG4	Cspg4	5,610309824	1,44826E-48	2,309358686	8,68296E-07
SLCO2A1	Slco2a1	5,374133213	5,73149E-19	2,051727518	0,022058307
FAM162B	Fam162b	8,188314643	6,06537E-13	3,983249692	0,041888308
TBXA2R	Tbxa2r	3,825990391	1,79146E-25	2,818407072	1,21431E-10
KCNMB1	Kcnmb1	2,31381301	1,50696E-07	2,496991961	8,17934E-06
BTNL9	Btnl9	3,622797636	0,007775227	5,300408794	0,009745992
AURKB	Aurkb	2,375854134	0,001301105	5,606890742	1,21283E-09
BCL6B	Bcl6b	7,92892908	1,96741E-44	2,338652895	0,00180216
COL1A1	Col1a1	3,352585079	2,17387E-11	4,345516491	4,39632E-14
CCR7	Ccr7	5,311782119	1,03524E-05	4,626177788	0,022020461
AOC3	Aoc3	7,949075281	4,32728E-37	2,290283645	0,00226779
HIGD1B	Higd1b	6,404330989	5,74168E-36	2,351057762	0,000105584
CD79B	Cd79b	2,918896576	0,000614328	2,828945508	0,021398415
BIRC5	Birc5	2,233813984	2,47548E-05	3,576253562	8,51488E-09
RRM2	Rrm2	2,304106206	4,36122E-06	3,435766532	3,88973E-09
BATF	Batf	2,964333734	2,91342E-09	3,430558923	5,66335E-05
EDARADD	Edaradd	6,220702978	3,21012E-11	2,848203966	0,018654953
NID1	Nid1	5,644033797	3,01196E-49	2,322644611	6,21082E-07
OGN	Ogn	2,01755801	0,000905107	2,17621333	0,004975583
HK3	Hk3	2,578725404	9,92109E-06	2,027808565	0,01524426

ITGA1	Itga1	2,429321534	5,84557E-15	2,022109525	3,40376E-08
RNASE1	Rnase1	3,536595008	7,75716E-10	4,693162526	2,32012E-05
ESCO2	Esco2	2,336040009	0,000540878	2,710945422	0,005703686
GPIHBP1	Gpihbp1	8,506362487	3,6146E-24	4,000980296	6,61459E-08
GTSE1	Gtse1	2,648082827	0,000196812	2,173733982	0,028434196
OLIG1	Olig1	3,505513624	0,000892646	3,331331771	0,015616599
RIPPLY3	Ripply3	2,424324219	0,017540114	2,426460427	0,029986403
ERG	Erg	7,365882117	6,09317E-80	2,444481363	8,16667E-07
MMP25	Mmp25	2,483062263	1,1221E-08	2,813809559	3,40086E-08
NDC80	Ndc80	3,219313488	1,6897E-11	3,217412021	1,48197E-07
PCDH12	Pcdh12	3,997797335	6,2772E-25	2,537917895	7,39904E-08
PIEZO2	Piezo2	2,95229404	1,24366E-15	3,291741683	5,30104E-14
CD248	Cd248	5,232806324	1,69727E-42	3,723270151	3,39212E-17
ACTA2	Acta2	2,35497604	2,30354E-08	3,676160081	4,28356E-15

Suppl. Table 2: Phylogenetically conserved mediators of retinal neovascularization (RNV). Phylogenetically conserved human (Sym_human) and murine (Sym_murine) DEG with respective log2 fold change (log2FC) and adjusted p-value (p_adj) for the comparison in human and murine (OIR p17) samples.
